# Application of artificial neural network in daily prediction of bleeding in ICU patients treated with anti-thrombotic therapy

**DOI:** 10.1186/s12911-023-02274-5

**Published:** 2023-08-31

**Authors:** Daonan Chen, Rui Wang, Yihan Jiang, Zijian Xing, Qiuyang Sheng, Xiaoqing Liu, Ruilan Wang, Hui Xie, Lina Zhao

**Affiliations:** 1 Department of Critical Care Medicine, Shanghai General Hospital, Shanghai Jiao Tong University School of Medicine, No. 650 New Songjiang Road, Songjiang, Shanghai, 201600 China; 2 Deepwise Artificial Intelligence Laboratory, Beijing, China

**Keywords:** MIMIC-III, Bleeding risk, Machine learning, Anti-thrombotic therapy, Algorithm recurrent neural networks

## Abstract

**Objectives:**

Anti-thrombotic therapy is the basis of thrombosis prevention and treatment. Bleeding is the main adverse event of anti-thrombosis. Existing laboratory indicators cannot accurately reflect the real-time coagulation function. It is necessary to develop tools to dynamically evaluate the risk and benefits of anti-thrombosis to prescribe accurate anti-thrombotic therapy.

**Methods:**

The prediction model,daily prediction of bleeding risk in ICU patients treated with anti-thrombotic therapy, was built using deep learning algorithm recurrent neural networks, and the model results and performance were compared with clinicians.

**Results:**

There was no significant statistical discrepancy in the baseline. ROC curves of the four models in the validation and test set were drawn, respectively. One-layer GRU of the validation set had a larger AUC (0.9462; 95%CI, 0.9147–0.9778). Analysis was conducted in the test set, and the ROC curve showed the superiority of two layers LSTM over one-layer GRU, while the former AUC was 0.8391(95%CI, 0.7786–0.8997). One-layer GRU in the test set possessed a better specificity (sensitivity 0.5942; specificity 0.9300). The Fleiss’ k of junior clinicians, senior clinicians, and machine learning classifiers is 0.0984, 0.4562, and 0.8012, respectively.

**Conclusions:**

Recurrent neural networks were first applied for daily prediction of bleeding risk in ICU patients treated with anti-thrombotic therapy. Deep learning classifiers are more reliable and consistent than human classifiers. The machine learning classifier suggested strong reliability. The deep learning algorithm significantly outperformed human classifiers in prediction time.

## Introduction

Coagulopathy caused by excessive activation of the coagulation system is a vital mechanism of organ failure [1, 2]. High risk factors of coagulation activation were found in critically ill patients, such as atrial fibrillation, unstable coronary heart disease, tumor, long diaphysis fracture surgery, medical device implantation, etc. Anti-thrombosis guarantees the administration of life and organ support techniques such as blood purification and extracorporeal membrane oxygenation. Anti-thrombotic therapy is the basis of thrombosis prevention and treatment [3–5]. Therefore, anti-thrombosis is widely applied to critically ill patients.

Bleeding is the main adverse event of anti-thrombosis [6]. Coagulation monitoring and evaluation are complicated. There are many differences among each patient, including etiology, host response, organ function, and clinical interventions [7]. The existing laboratory examinations of clotting function are neither able to reflect coagulation nor to predict haemorrhage. Some specific diseases, such as severe liver dysfunction [8], will lead to significant abnormalities in coagulation test results, different from the actual state of hyper-coagulable [9–11]. Once a bleeding event occurs in patients treated with anti-thrombotic therapy, it is usually severe [12]. It generally led to prolonged hospitalization and life-threatening events. Mortality rates can be increased by 1.5-5 times [13], especially when intracranial haemorrhage occurs [13, 14]. Therefore, anti-thrombotic therapy requires daily assessment and timely adjustment. Critical patients will experience different pathophysiological changes, such as acidosis, hypothermia, shock, hypoxemia, hypoproteinemia, etc. These changes and other factors, such as the dosage of anticoagulant drugs, the patient’s weight, and hepatic and renal function, have an impact on anticoagulant therapy. The technologies of organ support, which may activate coagulation, cannot be separated from anticoagulants. Evaluation is challenging since existing laboratory indicators cannot accurately reflect the real-time coagulation function. Existing anti-thrombotic therapy guidelines, such as Anti-thrombotic Therapy and Prevention of Thrombosis, 9th ed: American College of Chest Physicians Evidence-Based Clinical Practice Guidelines [15] and American Gastroenterological Association-Canadian Association of Gastroenterology Clinical Practice Guidelines: Management of Anticoagulants and Anti-platelet Agents During Acute Gastrointestinal Bleeding and Endoscopy [16], are not applicable to ICU patients. Therefore, it is necessary to develop tools to dynamically evaluate the risk and benefit of anti-thrombosis in line with the pathophysiological and pharmacokinetic characteristics of ICU patients, to prescribe accurate anti-thrombotic therapy.

With the rapid development of artificial intelligence(AI) neural networks, there have been a number of studies applying traditional machine learning or deep learning algorithms to predict bleeding events, including predicting their risk based on the least absolute shrinkage and selection operator (LASSO) algorithm [17], using classification regression trees (CART) and other algorithms to predict the bleeding risk of anti-thrombotic therapy in patients with deep vein thrombosis [18] and so on. At present, there are a lot of long-term bleeding prediction models for single disease, such as new oral anticoagulant atrial fibrillation patients bleeding prediction model [19], but there is no daily predictive model for complex multifactorial concurrent antithrombotic therapy in ICU patients, so comparisons cannot be made. This study intends to use the deep learning algorithm of the recurrent neural network(RNN), RNNs can retain a hidden state or memory at each time step, passing information from previous time steps to the current time step, allowing for a better understanding and prediction of changes and trends in time series data [20], algorithm to develop a dynamic prediction model for adverse events of critically ill patients based on the Medical Information Mart for Intensive Care (MIMIC) dataset. In order to apply the new AI deep learning technology to establish a dynamic and individualized anti-thrombotic therapy for critically ill patients, to guide ICU physicians to timely implement anti-thrombotic measures, to reduce the incidence of adverse events such as bleeding and to improve clinical efficacy.

## Methods

### Database

This study was a retrospective study, all data were collected from the Medical Information Mart for Intensive Care III (MIMIC-III)(https://physionet.org/content/mimiciii/1.4/), which is a large, open-source, single-center critical care database that collected more than 50,000 patients’ information from Beth Israel Deaconess Medical Centre in Massachusetts, USA, from 2001 to 2012 [21, 22]. All experiments were performed in accordance with relevant guidelines and regulations (Declaration of Helsinki). This database includes patients’ basic information without names, diagnosis, laboratory test results, and other helpful information. The ethics committees of the Massachusetts Institute of Technology and Beth Israel Deaconess Medical Centre have approved the implementation of the project. Requirements for individual patient consent and an ethical approval statement were waived in Shanghai General Hospital because the project do no harm to humans, there’s no commercial interest, and did not impact clinical care and the data used in this database was anonymous. Researchers can apply for permission for free. The users, must pass a test to qualify to register for the database and be approved by MIMIC-III database administration staff. After passing a training course “Protecting Human Research Participants” on the website of National Institutes of Health (NIH), an author (Daonan Chen) was approved to extract data from this database for research purposes (certifcation number: 38,314,451). Since the latest MIMIC-IV database had no medical text content until the completion of this research and the medical text was necessary to diagnose bleeding in this study, the database version used in this research was MIMIC-III v1.4.

### Study population

Patients in the MIMIC-III database treated with anti-platelet or anti-coagulant drugs were included in this study. Anti-platelet and anti-coagulant drugs included aspirin, clopidogrel, ticagrelor, warfarin, rivaroxaban, dabigatran, heparin, enoxaparin, and fondaparinux. Patients who used these drugs for less than two days or had bleeding events on the first day of administration were excluded, and the remaining patients were included in the final study.

### Outcome definition

We used the bleeding events of medical text and the discontinued events of anti-platelet or anti-coagulant drugs to determine whether the patients were bleeding. For the bleeding events of the medical text, we referred to the judging criteria provided by Taggart’s article [23]. There were two situations considered bleeding events, including medical text on the day of drug withdrawal and on the day before. The time of bleeding events in the medical text was regarded as the final bleeding time.

### Data extraction

We collected clinical and laboratory indicators of all patients from MIMIC-III database(https://physionet.org/content/mimiciii/1.4/) to build the model, including basic information (age, gender, comorbidities), vital signs (respiratory rate, blood pressure, heart rate, body temperature, pulse oxygen saturation), laboratory data (blood gas analysis, routine blood test, liver, and kidney function results, coagulation test and so on), and the type of anti-platelet and anti-coagulant drugs. In this study, we referred to Darzi et al.‘s review [24] and related research for the selection of relevant features and weights, which will help avoid selection bias and improve the robustness and generalization ability of the model. The weight of the indicators was divided into four levels: the first level: clotting time indicators, TEG, platelets, the dosage and route of use of anti-thrombotic drugs, etc.; The second level (physiological indicators that might affect the coagulation function and the metabolism and effects of anti-thrombotic drugs): liver and kidney function results, blood gas analysis, fluid load, albumin, et.; The third level (other risk-factor indicators in traditional prediction models): age, gender, past history, hormonal drugs use, etc.; The fourth level: other remaining indicators [25].

### Statistical analysis

We used Python (3.6), R (3.6), and PyTorch (1.7) for model building and analysis. Deepwise & Beckman Coulter DxAI platform (https://dxonline.deepwise.com) was used for analysis. Because continuous variables were not normally distributed, we used the median and interquartile range to express them in order to compare the basic information of different datasets. Categorical variables were expressed as the number of events and the percentage of events to the total events, using the Chi-square test to compare different datasets. Since the previous analysis showed that the proportion of bleeding events and non-bleeding ones was too small, we selected patients from the bleeding group who had non-bleeding events for at least one day before bleeding events and randomly selected 1,000 patients from the non-bleeding group for the final study. In the non-bleeding group, patients were randomly divided into the training set, validation set, and testing set according to the ratio of 8:1:1 for model construction. In the bleeding group, the patients were randomly divided into training: validation: testing set at the ratio of 8:1:1. In this research, each case corresponded to a unique icustay_id, and each icustay_id was distinct, representing a single ICU admission record for a patient. The training, validation, and testing datasets for the model were kept separate, and there was no overlap between them.

**Fig. 1:**
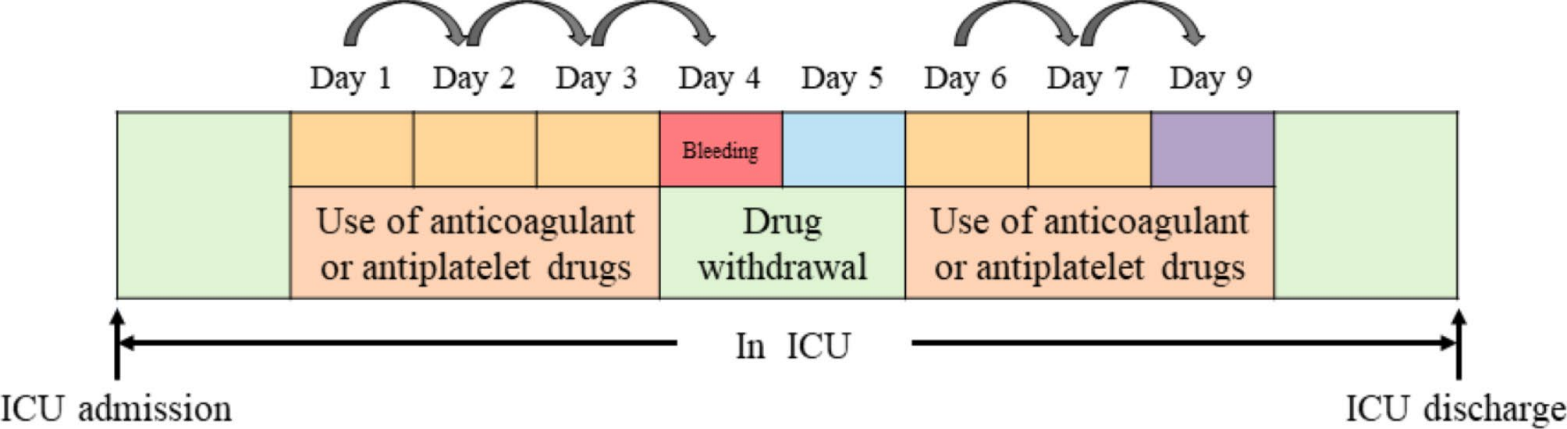
Dynamic prediction of bleeding event model: when the patient starts using anticoagulant or anti-platelet drugs, the model will predict and evaluate the risk of bleeding the next day based on daily indicators. If the patient has a bleeding event and the drugs are withdrawing, the model will stop, and when the treatment restarts, the prediction will be restarted

For bleeding patients, we used the data from the beginning of the drug use to the day before bleeding to train the model to predict whether there was bleeding on the next day. For non-bleeding patients, we used the data from the beginning of the drug use to the day before the drug withdrawal to train the model. We hoped that the final model could use the data from the previous few days to predict whether the patient would bleed the next day in order to implement antithrombotic measures (Fig. 1).

We conducted explorations with various RNN methods, eventually utilizing Long Short-Term Memory (LSTM) and Gated Recurrent Unit (GRU) architectures to build our model. Our model consists of a deep sequence model, which includes one or two layers of LSTM or GRU, and a linear layer. Patient clinical information, collected over multiple dates, was normalized using Z-score normalization and then fed into the model. The Z-score normalization is defined as $z=\left(x-\mu \right)/\sigma$, where $x$ represents the raw feature values, $\mu$ is the mean value of $x$, and $\sigma$ is the standard deviation of $x$. Given that the number of patient visits was not consistent, we adjusted sequences to a consistent length of 80. For sequences with fewer than 80 data points, we employed zero padding to reach this length. For sequences exceeding 80 data points, we truncated the sequence to fit the specified length.

The data was first passed through LSTM or GRU to generate 128-dimensional features, which were then inputted into the linear layer to output the probability of patient bleeding. The model’s parameters were updated through binary cross-entropy loss and the Adam optimizer. During the model training process, we determined the optimal hyperparameters (specifically, learning rate and batch size) via grid search, using the Area Under the Curve (AUC) metric on the validation set for verification. The final model was trained over 10 epochs with a batch size of 32 and a learning rate of 1e-3. Additionally, we masked the gradient at padding value positions to prevent missing data from contributing to model training.

We evaluated different models using the receiver operating characteristic (ROC) curve and area under the curve (AUC). We also used survival curves to compare the prognosis of bleeding and non-bleeding patients. Additionally, we compared the performance of each trained deep learning model with human evaluations on different metrics: accuracy, F1-score, precision, and recall. Accuracy and corresponding 95% CI were also compared. Inter-rater reliability (IRR) was carried out to measure to what extent different classifiers have the same result. Reliability does not measure whether classification matches the ground truth, only whether different classifiers agree on the same classification. k > 0.4 is a weak agreement, k > 0.6 is a moderate agreement, and k > 0.8 is a strong or almost perfect agreement [26]. In this study, p < 0.05 was considered statistically significant.

## Results

### Baseline characteristics

**Fig. 2:**
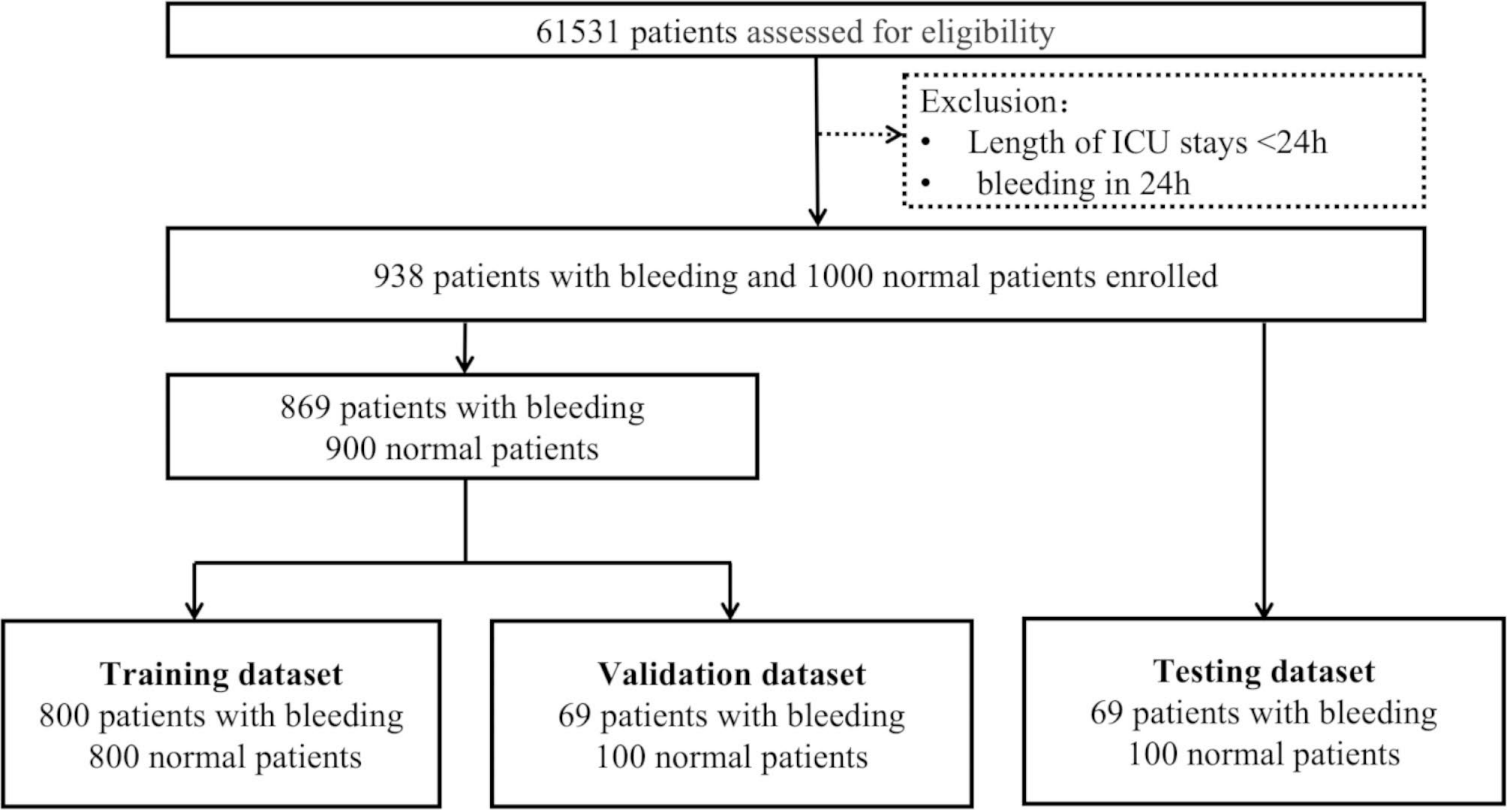
Flowchart of dataset construction

61,531 ICU patients were screened from the MIMIC-III database in this research. 30,270 patients treated with anticoagulant and anti-platelet agents remained after exclusion of less than 24-hour ICU stays and bleeding on the first day of admission. There were 29,332 non-bleeding patients (96.9%) and 938 bleeding patients (3.01%). All of the 938 patients were incorporated into the study, and at the meantime, 1000 non-bleeding patients were randomly extracted. Totally, 1938 patients of the two groups were eventually brought into the study. 800 patients were respectively selected from the two groups to establish a training set, and the rest 338 patients were divided into a test and validation set at the ratio of 1:1(Fig. 2). There was no significant statistical discrepancy among the three sets in baseline, which included gender, age, underlying diseases, and so on (Table 1).

**Fig. 3:**
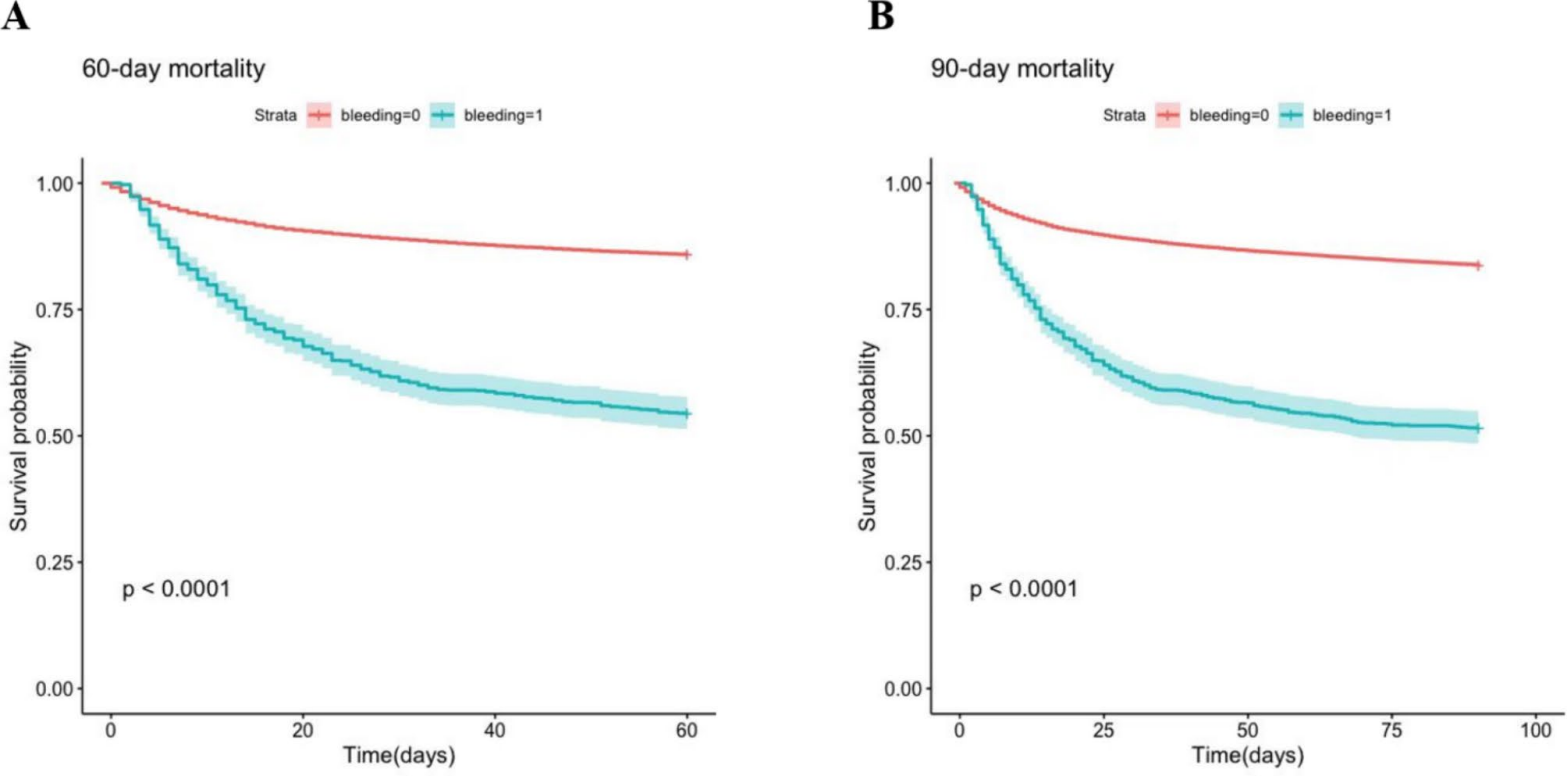
Survival curves at 60 and 90 days in patients with and without bleeding

**Table 1:** Demographic data of patients

	Training set (N = 1600)	Validation set (N = 169)	Testing set (N = 169)	*P* value
Age (median [IQR])	68.69 [55.50, 79.05]	71.38 [59.00, 79.76]	69.13 [57.76, 78.33]	0.442
Male (%)	918 (57.4)	98 (58.0)	88 (52. 1)	0.400
Congestive heart failure (%)	58 (3.6)	8 (4.7)	6 (3.6)	0.764
Cardiac arrhythmias (%)	6 (0.4)	0 (0.0)	0 (0.0)	0.530
Valvular disease (%)	59 (3.7)	10 (5.9)	2 (1.2)	0.068
Peripheral vascular disease (%)	57 (3.6)	8 (4.7)	1 (0.6)	0.078
Other neurological disease (%)	15 (0.9)	1 (0.6)	4 (2.4)	0.182
Hypertension (%)	8 (0.5)	0 (0.0)	1 (0.6)	0.640
Chronic pulmonary disease (%)	11 (0.7)	0 (0.0)	3 (1.8)	0.145
Diabetes uncomplicated (%)	6 (0.4)	0 (0.0)	0 (0.0)	0.530
Hypothyroidism (%)	1 (0.1)	0 (0.0)	0 (0.0)	0.900
Renal failure (%)	2 (0.1)	0 (0.0)	0 (0.0)	0.809
Liver disease (%)	18 (1.1)	0 (0.0)	0 (0.0)	0.147
Peptic ulcer (%)	1 (0.1)	0 (0.0)	0 (0.0)	0.900
AIDS (%)	7 (0.4)	2 (1.2)	0 (0.0)	0.259
Lymphoma (%)	9 (0.6)	0 (0.0)	0 (0.0)	0.385
Solid tumor (%)	55 (3.4)	8 (4.7)	3 (1.8)	0.320
Rheumatoid arthritis (%)	4 (0.2)	0 (0.0)	0 (0.0)	0.655
Coagulopathy (%)	1 (0.1)	0 (0.0)	0 (0.0)	0.900
Alcohol abuse (%)	3 (0.2)	1 (0.6)	1 (0.6)	0.412

### 60- day and 90-day mortality rate

The number of death patients in non-bleeding and bleeding groups was respectively 427 and 4156 (14.17% vs. 45.52%), and the number of death patients in 90-day was 454 and 4757 (16.22% vs. 48.40%). Kaplan-Meier was used to draw the survival curve in 60 and 90 days, and the graph showed the mortality of both in the bleeding group is remarkably higher than that in non-bleeding group (Fig. 3).

### Model performance comparisons

**Fig. 4:**
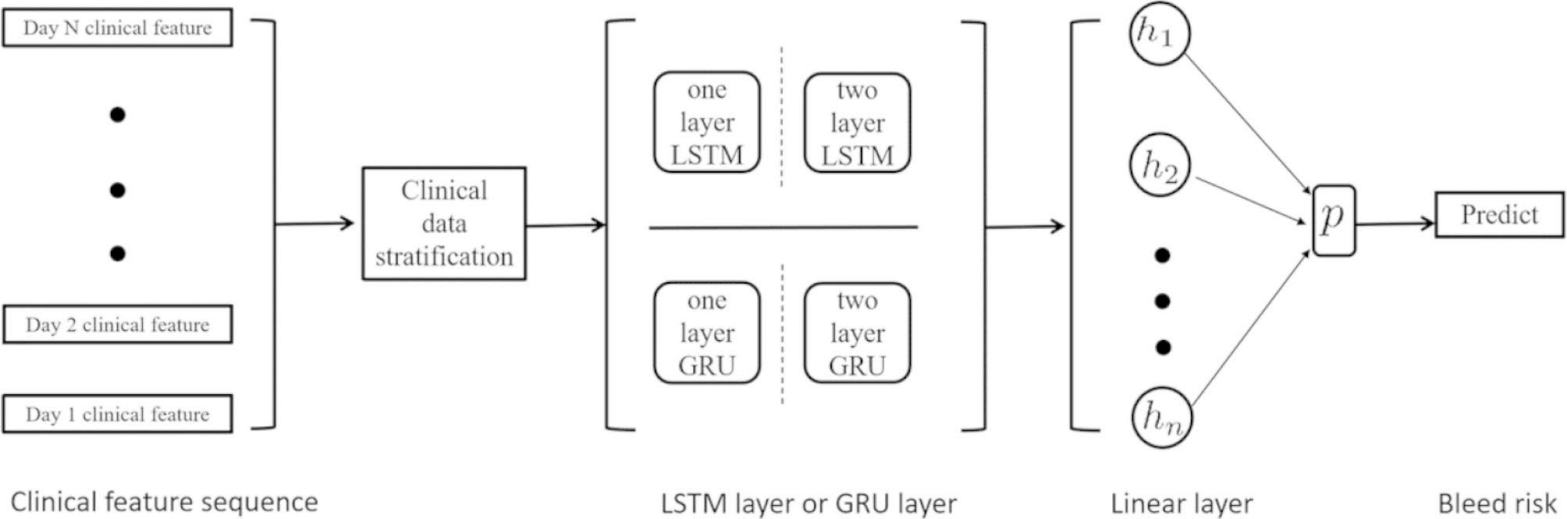
Schematic illustration of model development

Clinical parameters from the beginning of anti-thrombotic drugs administration to bleed, transfer, and discharge of all patients were collected, and RNN was used to establish haemorrhage prediction model of anti-thrombotic therapy. The model was composed of a linear layer and a deep sequence model, including 1 or 2 layers of LSTM or GRU. To be specific, the clinical data sequence, which included the clinical test data for multiple days of the patients, was input into the model. The data were first passed through LSTM or GRU to generate 128-dimensional features and then were input into the linear layer to output the probability value of bleeding. Cross-entropy loss was adopted in the model, and the learning rate algorithm was optimized with Adam. The ratio of prediction probability to the pre-determined probability of bleeding was 0.5, and it was predicted to bleed when the ratio was over 0.5 (Fig. 4).

**Fig. 5:**
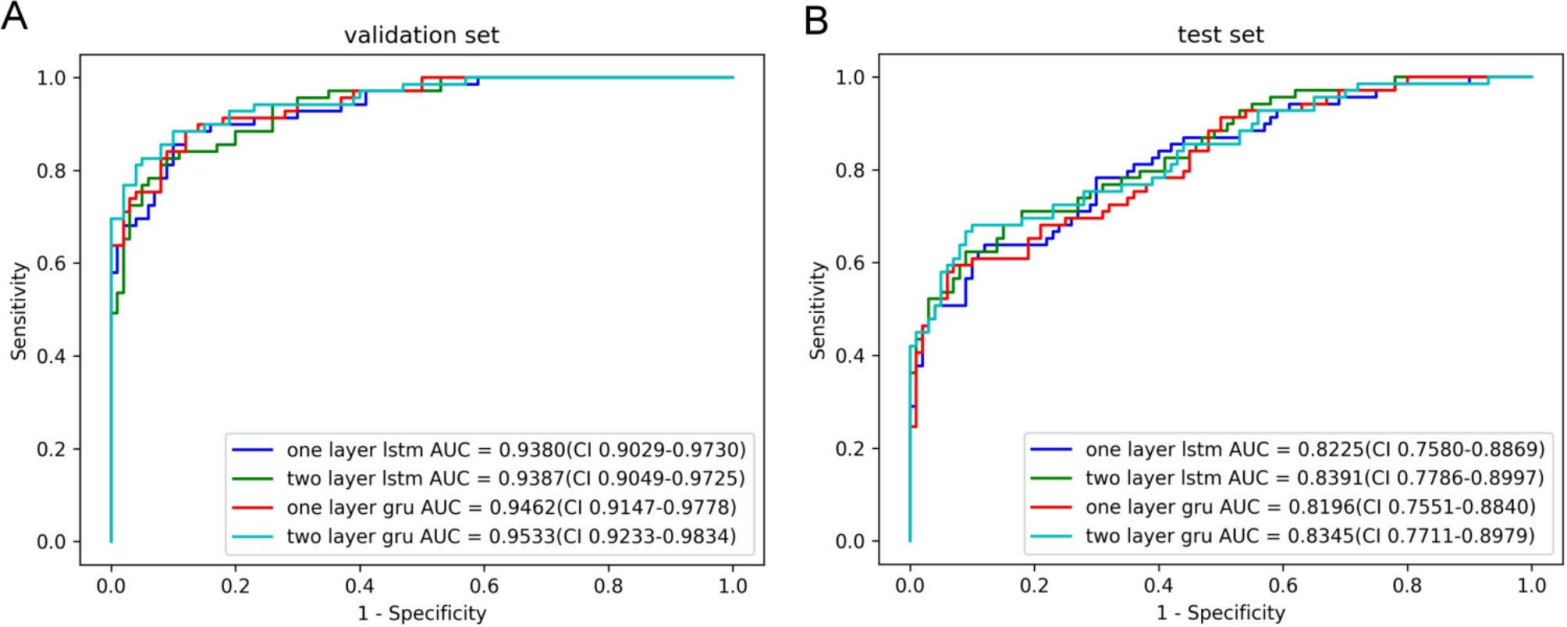
Model performance in different patient cohorts. A: ROC curve of the validation set. B: ROC curve of the test set

ROC curves of the four models in the validation set and test set were drawn, respectively (Fig. 5). One-layer GRU in the validation set possessed a larger AUC (0.9462; 95%CI, 0.9147–0.9778), and the sensitivity and specificity of the prediction model were 0.8261 and 0.9100, respectively. Analysis was conducted among the 169 patients in the test set, and the ROC curve showed that two-layer LSTM was superior to one-layer GRU. The AUC of the former was 0.8391 (95%CI, 0.7786–0.8997), and the sensitivity and specificity were, respectively, 0.6232 and 0.9100. However, one-layer GRU had higher specificity (sensitivity 0.5942, specificity 0.9300), and the AUC was 0.8196 (95%CI, 0.7551–0.8840). The sensitivity and specificity of the four prediction models were summarized in Table 2.

**Table 2:** Performance of the final models in validation and text set

Model	Validation set (MIMIC-III)					Testing set (MIMIC-III)			
	AUC	Youden	Sensitivity	Specificity		AUC	Youden	Sensitivity	Specificity
One layer LSTM	0.9380	0.7641	0.8841	0.8800		0.8225	0.5177	0.6377	0.8800
Two layer LSTM	0.9387	0.7361	0.8261	0.9100		0.8391	0.5332	0.6232	0.9100
One layer GRU	0.9462	0.7641	0.8841	0.8800		0.8196	0.5242	0.5942	0.9300
Two layer GRU	0.9533	0.7841	0.8841	0.9000		0.8345	0.5812	0.6812	0.9000

### Compared with manual verification

Here we demonstrate the effectiveness of deep learning in the daily prediction of bleeding in ICU patients treated with anti-thrombotic drugs. Using the GRU model trained on general bleeding classification, we match the performance of at least 5 doctors in the ICU tested. Reliability measures are suggested to measure whether different classifiers agree on the same classification result. It is measured by Fleiss’ k as our data contains more than 2 classifiers per set. K has an upper limit of 1, and negative values of k imply disagreement beyond what would be expected by chance alone. Kappa is suggested to evaluate the interrater reliability with the following scale: k > 0.4 is a weak agreement, k > 0.6 is a moderate agreement, and k > 0.8 is a strong or almost perfect agreement.

The Fleiss’ k of junior clinicians, senior clinicians, and deep learning classifiers is 0.0984, 0.4562, 0.8012, respectively (Table 3). Junior doctors refer to resident doctors, who are still in the stage of standardized training for resident physicians and specialist physicians. Senior doctors refer to doctors who are capable of independently handling their specialty and who guide the work of junior doctors. The clinicians involved, who did not participate in the performance review, individually judged all data consistent with the machine-validated dataset to determine whether there was bleeding. The reliability results indicate that deep learning classifiers are more reliable than human classifiers.

The time that each classifier used was also compared. The Junior clinicians utilized an average of 77.50 min; the senior clinicians spent an average of 53 min classifying the events. All the deep learning classifiers generated prediction results within 1 min.

**Fig. 6:**
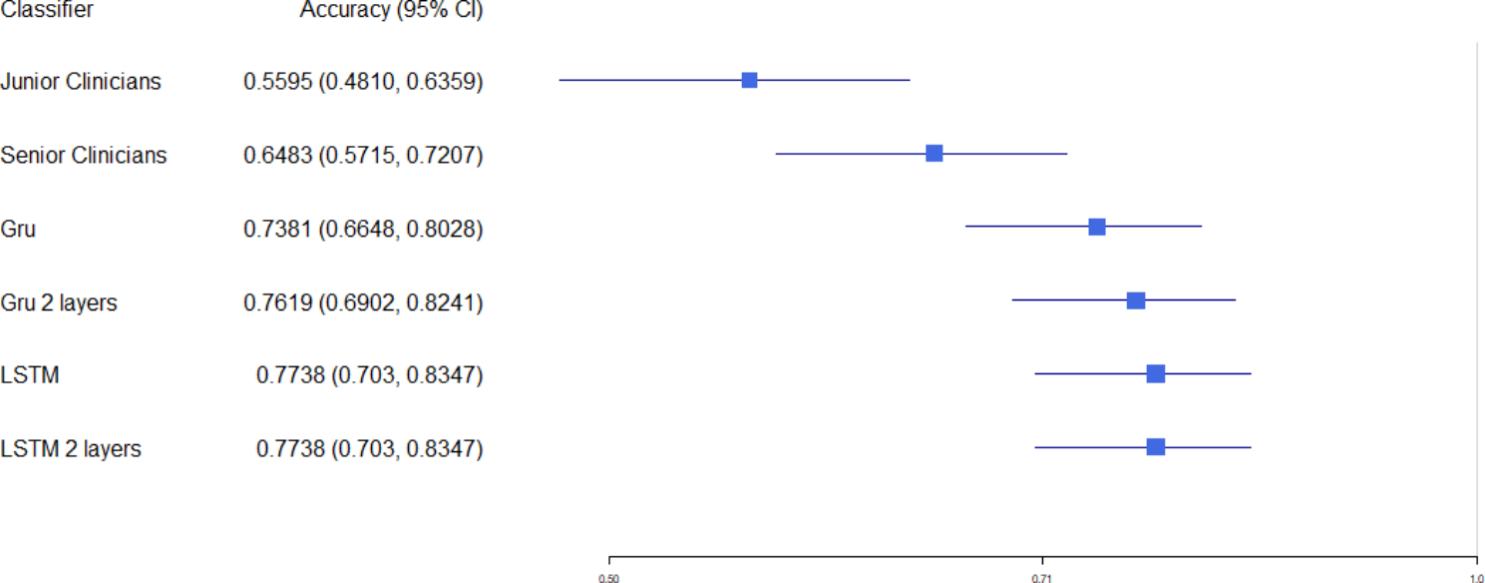
The accuracy of the model and clinicians

**Fig. 7:**
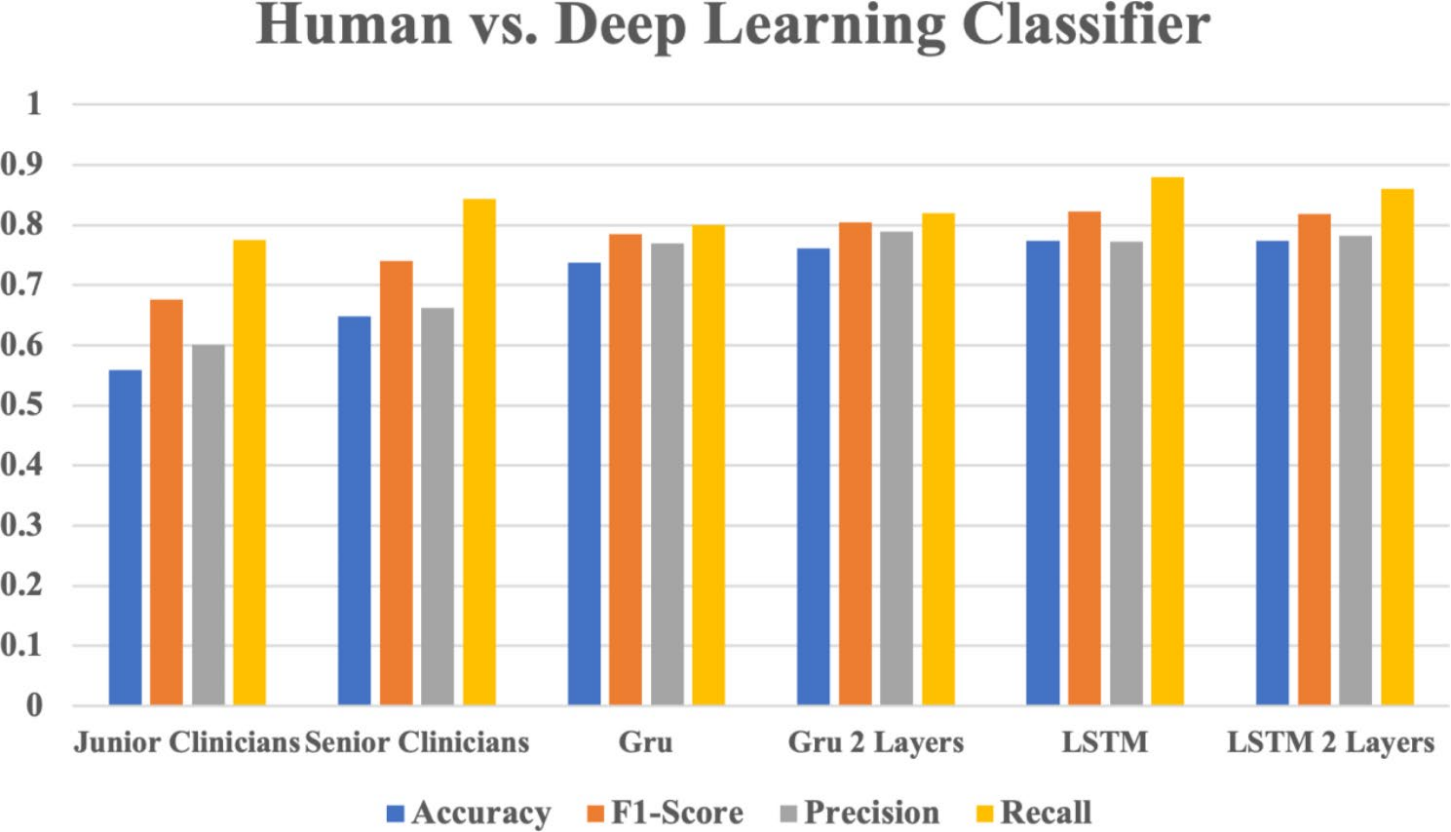
The overall performance of deep learning classifiers versus human detection on the bleeding events

The accuracy of all deep learning classifiers is above 0.7, which is significantly higher than that of senior clinicians (0.6488) and junior clinicians (0.5595) (Fig. 6). The confidence intervals of junior clinicians and deep learning classifiers do not overlap. Figure 7 illustrates the overall performance of deep learning classifiers versus human detection of bleeding events. Among the classifiers, the deep learning classifiers outperformed human classifiers in accuracy, F1-score and precision, and obtained a similar recall.

**Table 3:** The inter-rater reliability

	k	95% CI
Junior Clinicians	0.0984	(-0.0528, 0.2496)
Senior Clinicians	0.4562	(0.3689, 0.5435)
Machine Learning Classifiers	0.8012	(0.7395, 0.8629)

## Discussion

To our knowledge, this is the first attempt to apply neural network models for the daily prediction of bleeding in ICU patients treated with anti-thrombotic drugs. Our study developed four variants of dynamic neural network models, providing an accurate predictive tool for bleeding prediction in ICU patients during anti-thrombotic therapy. In our study, we reconfirmed that bleeding events worsen the clinical outcomes of patients treated with anti-thrombotic therapy in ICU. According to the MIMIC-III database, the incidence of haemorrhage in patients with anti-coagulation therapy during the stay of ICU was 4.77%. As shown in Fig. 4, the bleeding events can lead to a higher mortality rate (both 60-day and 90-day), and longer length of hospital/ICU stay. As a result, early identification of bleeding risks during anti-thrombotic therapy is of great importance.

ICUs treat a heterogeneous group of patients whose risk of bleeding can vary widely and change quickly. Hence the early identification of haemorrhage can be quite difficult. Currently, there is a lack of reliable tools for the dynamic prediction of bleeding among these patients. Our study demonstrated that the family of RNN, such as LSTM and GRU, can predict bleeding with high accuracy.

After we weighed the input values according to clinical practice, GRU was applied to build models on time-updated data. RNNs are called recurrent because they perform the same task for every element of a sequence, with the output being dependent on the previous computations. Another way to think about RNNs is that they have a “memory” that captures information about what has been calculated so far. Theoretically, RNNs can use information in arbitrarily long sequences, but in practice, they are limited to looking back only a few steps. RNNs employing either of these recurrent units have been shown to perform well in tasks that require capturing long-term dependencies [27]. Thus, we used the RNNs for training dynamic models to identify trends in patients’ conditions and predict bleeding events. The most used type of RNNs is LSTM, which can be useful for clinical measurements because they carefully tune the information passed between subsequent time iterations of the model. Advantages of LSTM over regression models include the ability to generate multiple predictions with the first data input and the ability to combine features in more complex ways to model changes over time [28]. Another type of recurrent unit, which we refer to as a gated recurrent unit (GRU), was proposed to make each recurrent unit adaptively capture dependencies of different time scales. Similarly to the LSTM unit, the GRU has gating units that modulate the flow of information inside the unit, however, without having a separate memory cell [27].

In this study, we developed four variants of RNN models that can identify patients with a high risk of bleeding and help the clinical decision-makers to adjust anti-thrombotic drugs. As shown in Fig. 3 and Table II, our models had equally predictive performance, demonstrating that RNN models based on big data have good generalization capability. The neural network models were compared with manual verification. The Fleiss’ k of junior clinicians, senior clinicians, and machine learning classifiers is 0.0984, 0.4562, 0.8012, respectively. The time used to generate the prediction results using a deep learning classifier is significantly lower than clinicians, which generated results within one minute.

We envision the future of care for all patients with anti-thrombotic therapy in ICU to be enhanced by customized machine learning decision support tools that will provide both initial risk stratification and ongoing risk assessment to adjust treatment at the right time for the right patient. By using a dynamic risk assessment, bleeding events could be identified early and prevented as early as possible, which reduced the risk of mortality in these patients. It is necessary to improve the positive predictive rate. There are also such studies, the EPIC sepsis model has been deployed in hundreds of hospitals in the United States, but the positive predictive value is 12% [29], long-term false positive prediction alarms can lead to alarm fatigue. How to improve the positive predictive value of the model is also a problem that we need to solve further. Suitable alarms can better serve patients [30]. With the advancement of mathematical algorithms, it may also further optimize our model. We need to constantly learn, update and iterate. For example, using an unaligned method based on multiple convolutional neural networks (CNN) and position-specific scoring matrix (PSSM) contours to identify coiled-coil protein models to further improve prediction accuracy [31].The most important thing is that the model needs to be deployed to the electronic medical record system, and the doctor should be notified after the alarm is triggered, so that the doctor can evaluate and adjust the treatment plan [32]. The optimized model applied to clinical practice can remind clinical doctors to pay attention to high-risk bleeding patients after antithrombotic therapy, discover abnormalities as early as possible, and adjust medication in time. We hope to improve patient outcomes in the future.

Several limitations of this study should be considered. Firstly, due to the limited number of bleeding patients in the database, additional iterations are required to enhance accuracy. Secondly, the MIMIC-III database lacks certain indicators. As such, it will be necessary in future studies to establish a prospective dataset of anti-thrombotic therapy in ICU patients. Thirdly, At present, our model is being conducted such a preliminary exploratory study. Subsequently, based on this model, further external validation, training and optimization of the model will be carried out with a large sample size. The prediction of bleeding outcomes in patients may vary among different populations and genders. Therefore, we need a larger dataset to avoid data bias. External validation work similar to the one reported in this literature, such as a prediction model for avoiding the occurrence of adverse reactions when drugs and food are used together [33]. Finally, the interpretability of recurrent neural networks can be challenging due to their inherent complexity, particularly in relation to time step folding. In our future work, we aim to explore potential methods to improve model interpretability. These may include the application of the DeepLIFT algorithm [34] to rank the feature importance of the 2-layer LSTM, or the implementation of a simpler linear model, such as logistic regression, to model the input and output of the LSTM and explain the feature contribution.

Due to the small number of bleeding patients in the database, iterations are needed to improve accuracy. Some indicators are missing from the MIMIC-III database. In the future, it is necessary to establish a prospective data set of anti-thrombotic therapy in ICU patients. In addition, the model needs to be further verified in external data.

## Conclusions

In conclusion, we present the first application of recurrent neural networks for the daily prediction of bleeding risk in ICU patients treated with anti-thrombotic therapy using the MIMIC-III database. Deep learning classifiers are more reliable and consistent than human classifiers. The machine learning classifier suggested strong reliability. The deep learning algorithm significantly outperformed human classifiers in prediction time.

## Data Availability

The datasets used and/or analyzed during the current study are available from the corresponding author on reasonable request.

## Abbreviations

ICU: Intensive care unit
AI: Artificial intelligence
LASSO: Least absolute shrinkage and selection operator
CART: Classification regression trees
RNN: Recurrent neural network
MIMIC: Medical Information Mart for Intensive Care
MIMIC-III: Medical Information Mart for Intensive Care III
NIH: National Institutes of Health
LSTM: Long short-term memory
GRU: Gated recurrent unit
ROC: Receiver operating characteristic
AUC: Area under the curve
IRR: Inter-rater reliability
